# Lean management in a liaison psychiatry department: implementation, benefits and pitfalls

**DOI:** 10.1192/bjb.2019.64

**Published:** 2020-02

**Authors:** Lauren Alexander, Susan Moore, Nigel Salter, Leonard Douglas

**Affiliations:** St Vincent's University Hospital, Dublin, Ireland

**Keywords:** Liaison psychiatry, quality improvement, lean management

## Abstract

**Aims and method:**

To apply process mapping, a component of lean management, to a liaison psychiatry service of an emergency department. Lean management is a strategy that has been adapted to healthcare from business and production industries and aims to improve efficiency of a process. The process consisted of four stages: individual interviews with stakeholders, generation of process maps, allocation of goals and assessment of outcomes.

**Results:**

There was a significant reduction in length of stay of psychiatric patients in the emergency department (median difference: 1 h; *P* = 0.015). Five of the six goals were met successfully.

**Clinical implications:**

This article demonstrates a management intervention that successfully reduced length of stay in an emergency department. Further to the improvements in tangible (quantitative) outcomes, process mapping improved interpersonal relations between different disciplines. This paper may be used to guide similar quality improvement exercises in other areas of healthcare.

Lean management is a process that has been adapted for use in healthcare from business and production industries. Its usefulness has been demonstrated in medical settings, where it generates enhanced staff understanding and improved, coordinated delivery of care.^1–3^ Research indicates similar benefits in behavioural crisis units^4^ but its use in an emergency department liaison psychiatry setting has not been demonstrated. Although psychiatric patients comprise a minority of emergency department presentations, they require a disproportionate amount of time and resources, which can frustrate emergency department staff and cause negative attitudes towards such patients.^5,6^ Myriad factors underlie this, such as lengthy waiting times, interpersonal difficulties and procedural ambiguity. These factors are often longstanding and resistant to change, but lean management processes, when executed appropriately, are an accessible and effective way of effecting meaningful change.

Prolonged length of stay was a recurrent source of contention and discontent in this emergency department before the process was undertaken. Boarding or lodging of psychiatric patients awaiting admission to psychiatric units is common and, for various reasons, these patients spend longer in the department than their medical and surgical counterparts.^7^ The requirement for ‘medical screening’ is contributory, but avoidable non-clinical factors, such as health insurance or lack of transport, are known to play a significant role.^8^ In addition to straining resources, patients who spend longer in an emergency department are more likely to suffer adverse outcomes or incidents, such as medication errors.^9^

Workplace incivility is a further stressor that is reportedly commonplace in emergency departments,^10^ and is compounded by the phenomenon of ‘silo working’, whereby different departments operate in isolation from each other.^11^ In addition to contributing to an unpleasant work environment, interpersonal conflicts interfere with provision of collaborative and efficient care. Efficient and coordinated delivery of care is essential to optimising the quality of treatment provided.^12^

## Aims

- To execute and describe a quality improvement process consisting of:
  - mapping the psychiatric patient's journey through the emergency department, using data from all involved healthcare professionals (stakeholders)
  - identifying weaknesses in the system, such as duplication of work, role confusion, communication errors and unnecessary delays
  - (iii) generating immediate and short-, medium- and long-term goals to improve service provision.
- To evaluate and quantify outcomes of the quality improvement process, including:
  (i) length of stay of patients in the emergency department before and after the quality improvement intervention
  (ii)  attainment of goals after intervention.

## Method

### Setting

This quality improvement exercise was executed in the emergency department of an urban university tertiary-referral hospital, processing in excess of 55 000 patients per year. This is a 24-hour emergency department that receives on average 29 psychiatric referrals per week. Mental health assessments are carried out by the consultation liaison psychiatry service during working hours and on-call psychiatry out-of-hours.

The process was executed in four stages:

- stage I – stakeholder interviews
- stage II – generation of process maps
- stage III – interdisciplinary meeting
- stage IV – assessment of outcomes.

Over a period of 3 months, we conducted interviews with 11 staff working in the emergency department.

### Selection of participants

The proposal for this project was submitted at an in-house Emergency-Psychiatry Management Meeting. We applied to interview 11 stakeholders – staff members from different disciplines who are routinely involved in providing care for mentally ill patients in the emergency department. They were: one emergency department triage nurse, one emergency department nurse, two emergency department doctors, one liaison psychiatry registrar, two liaison psychiatry nurse specialists, one psychiatry registrar on-call, one social worker, one healthcare assistant and one security staff. The data were to be collected by a senior member of the psychiatry team not routinely involved in first-line care in the emergency department.

### Ethical approval

Exemption from ethical approval was granted by the Ethics Committee in St Vincent's University Hospital, Dublin, on the basis that there was no direct patient involvement in this study.

### Stage I: stakeholder interviews

The purpose of stage I interviews was to elicit details of individual staff members' management of psychiatric patients in general, in order to map a generic template of the interaction, from beginning to end, between psychiatric patients and specific disciplines, such as social work.

Duration of interviews was 40–60 min, concluding when no new data were being generated.

In an individual face-to-face meeting, the stakeholder was invited to describe their involvement with psychiatric patients in the emergency department, beginning from the point at which they first become aware of the patient and concluding with their last contact with the case. The participants were asked to describe interactions with psychiatric patients in general, rather than specific issues that had arisen with individual patients.

The purpose of the interview (stage I) was explained to each participant. They were informed that they would be invited to participate in stages II and III at a later date. They were advised that the interviewer would redirect them if they began to engage in stage II or III discussion.

Participants were first asked open questions, such as ‘Tell me about your first contact with a psychiatric patient in the emergency department’, followed by more closed questions, such as ‘How long does this component take?’ Further questions were raised to identify specific weaknesses, such as duplication of work, role confusion, communication errors and unnecessary delays.

To reduce bias from the interviewer, the participant was allowed to speak without interruption except when redirection or clarification was required. Participants were redirected back to the routine care pathway if they began to discuss problem-solving.

### Stage II: generation of process maps

A working flow diagram of each participant's involvement was drawn up during the interview and the participant was asked to make any comments or changes before the meeting ended.

**Fig. 1:**
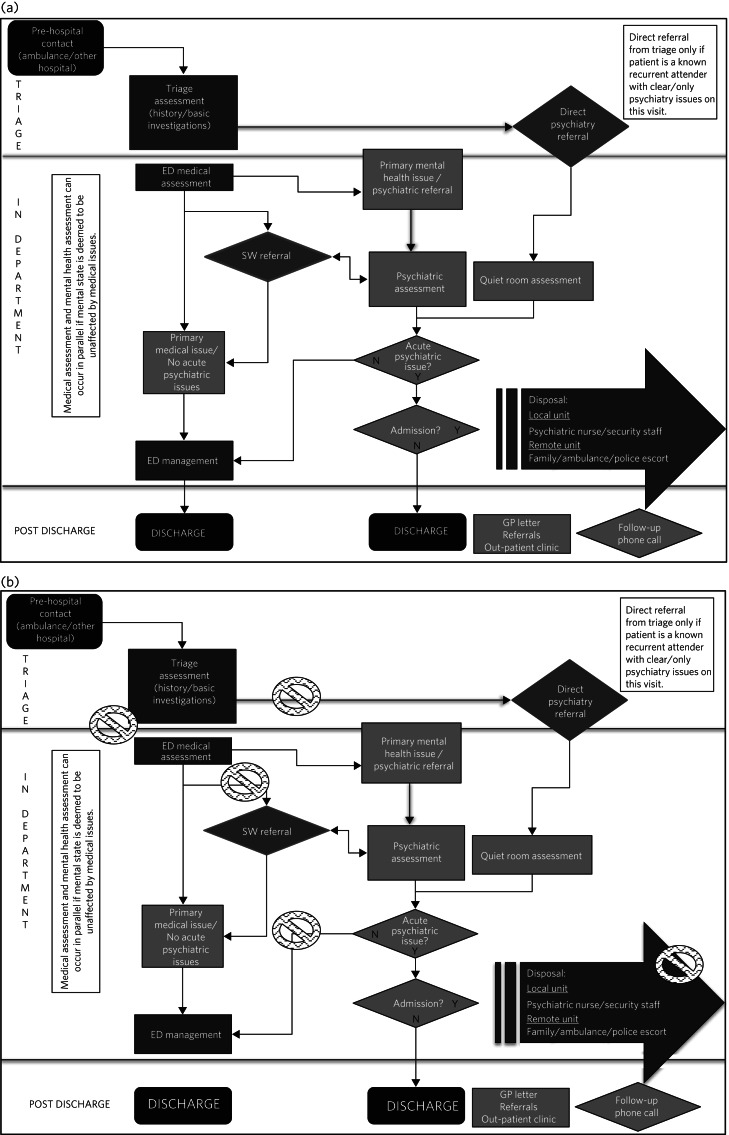
The overview process map, detailing the pathway of psychiatric patients through the emergency department. (a) Baseline map; (b) map with problem areas superimposed (marked by ‘no access’ symbol).

ED, emergency department; SW, social work; GP, general practitioner.

**Fig. 2:**
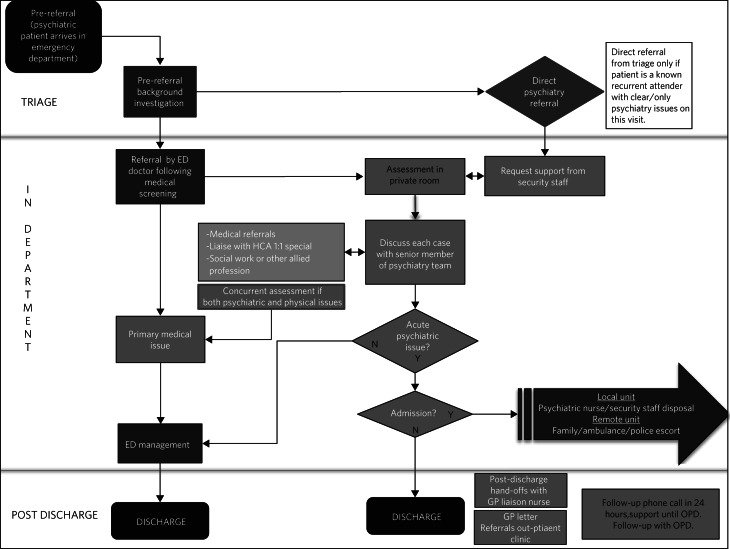
An individual process map representing the role of the liaison psychiatry nurse specialist: 3–4 h pathway of patient assessment and management in the emergency department (ED).

HCA, healthcare assistant; OPD, out-patient department; ED, emergency department; GP, general practitioner.

The information was transformed into an overview process map showing the patient journey and maps representing the role of each individual stakeholder (Figs 1 and 2).

### Stage III: post-interview stakeholder focus group and goal-setting

All stakeholders and heads of departments (medical, nursing, liaison psychiatry, social work, security) were invited to participate in a focus group. The focus group consisted of ten individuals.

The overview (integrated) map and individual maps (Figs 1 and 2) were scrutinised for weaknesses or pinch points. No solutions were suggested or elicited at this stage. Weaknesses were itemised into four groups: role confusion, duplication of work, unnecessary delays and communication deficits. A problem list was collated and distributed. Stakeholders were asked to categorise items according to priority (1, low priority; 2, medium priority; 3, high priority) and to mark a timeline for implementation (immediate, less than 1 month, more than 1 month). After consensus was reached on target areas (items that scored >20), stakeholders were invited to generate potential solutions. Responsibility for each target area was allocated (Table 1).

**Table 1:** Results of six targeted areas for change, including action prescribed, individual assigned and projected timeline

Proposed improvement	Weakness targeted	Action	Person responsible	Timeline	At 6-month follow-up^a^
Efficient handover after psychiatric assessments	i, ii, xiii, xiv	Designated emergency department staff members (team leaders A and B) to be the contact for psychiatry staff about all psychiatric patients in the emergency department	Psychiatry consultant to inform psychiatry staff; emergency department clinical nurse manager to inform emergency department nurses	Immediate	Target met on projected timeline
Emergency department staff access to liaison psychiatry team at morning shift-change	i, ii, iii, xiii, xiv, xvii	Priority discussion for emergency department nurse manager or social worker or emergency department doctor at liaison psychiatry handover meeting at 09.00 h	Psychiatry consultant to restructure handover meeting	Immediate	Target met on projected timeline
Refine referral pathway (emergency department to psychiatry)	v, vii, viii, ix, x, xi	Default referral to psychiatry is by emergency department medical staff, not triage or emergency department nurse. MITT to reflect this	Emergency department consultant to change MITT protocol. Emergency department nurse manager to inform triage nursing staff.	Immediate	Target met on projected timeline
		In limited circumstances, as defined, direct referrals are possible	Emergency department and psychiatry consultants to agree criteria for direct referral pathway between emergency department and psychiatry	Immediate to 1 month	Target met on projected timeline
Define medical screening	vii, x, xi	Emergency department and psychiatry consultants to collaborate on medical screening requirements	Emergency department and psychiatry consultants	1 month	Target revised and excluded at clinical meetings (see section IV: attainment of outcomes)
Prevent or manage acute agitation in psychiatric patients	v, vi	Implementation of psychiatric medication chart for patients awaiting transfer to approved psychiatric unit. Psychiatry will prescribe ‘as required’ medication and give advice proactively and pre-emptively	Psychiatry consultants to inform psychiatric registrars	Immediate	Target met on projected timeline
Formalise the role of security staff	vi, xv	Clarity about legal obligations and safeguards in relation to restraint and detention	Psychiatry consultants to provide formal written guidance and training for security staff	1 month	Target met on delayed timeline (3 months)

MITT, Mental Illness Triage Tool.

a. Five of the six targets were met, one on a delayed timeline. One target was discarded at subsequent clinical meetings.

### Stage IV: assessment of outcomes

Outcomes were evaluated 6 months after completion of stage III. Outcomes were patient length of stay in the emergency department and attainment of goals (Table 1).

### Statistical analysis

Lengths of stay of samples of patients referred for psychiatric assessment were measured over a 3-month period before commencing the process (January–March 2017) and over a 3-month period 6 months after the intervention (January–March 2018). In total, 190 pre-mapping patients and 190 post-mapping patients were compared. Distribution of data was calculated using the Shapiro–Wilk test. Statistical significance was calculated using the Mann–Whitney test and effect size was calculated by the difference between median lengths of stay in the pre-mapping and post-mapping groups. This was a convenience sample that represents over half the total number of psychiatry consults in emergency department over the 3-month post-mapping period. This was a convenience sample that represents over half the total number of psychiatry consults over a 3-month period, chosen from cases that were labelled as psychiatry consults on the emergency department's patient-processing software.

## Results

### Stage I: stakeholder interviews

Each of the 11 interviewees described their involvement with psychiatric cases, which were mapped onto individual maps (Fig. 2). The difficulties encountered were also elicited and categorised into four problem areas and 17 targets (i–xvii).

1. Delays:
  - on-call psychiatry doctors reported delayed handover of updates from emergency department staff
  - (ii)emergency department nursing reported delayed handover from psychiatry team following assessments
  - (iii)social work reported that the patient stay was prolonged by delays in referral from emergency department and psychiatry staff
    (iv)social work reported that medical and psychiatric assessments were delayed by failure to assess patients in tandem with social work assessment.
2. Role confusion:
  1. the discipline responsible for the management of acute agitation (emergency department doctors or psychiatry doctors) was unclear
  2. the role of security staff in the care of psychiatric patients, in particular the statutory limits of their interventions, was unclear
  3. the medical discipline responsible for ‘medical screening’ (emergency department, psychiatry or general medical), and the definition of ‘medical screening’, were unclear
  4. it was unclear which medical discipline (emergency department or psychiatry) was considered to be the treating team of patients following completion of psychiatric assessment
  5. consequent to role confusion (viii), it was unclear which discipline was responsible for further referrals
  6. consequent to role confusions (vii) and (viii), it was unclear which discipline was responsible for further investigations, such as blood tests/ECG/urine toxicology.
3. Duplication of work:
  - the emergency department nurse and triage nurse both reported routinely making phone contact with psychiatry doctors to inform them of the arrival of psychiatric patients, in addition to the referral that was made by the emergency department doctor after their assessment; the expectation from these phone contacts was unclear
  - some disciplines reported carrying out overlapping assessments, e.g. social work and psychiatry.
4. Communication difficulties:
  - emergency department nurses reported a failure by psychiatry to keep emergency department staff updated on psychiatric management plans
  - psychiatry staff reported a difficulty identifying emergency department staff to receive updates
  - security staff reported failure of medical staff (psychiatry and emergency department) to update the security team, leading to a longer security intervention than needed in some cases
  - the healthcare assistant reported delayed updates after change of treatment plans, leading to a longer healthcare-assistant intervention (1:1 special) than needed in some cases
  - the social worker reported delayed referrals of child welfare issues to social work.

### Stage II: generation of process maps

Individual process maps (Fig. 2) and an overview process map (Fig. 1) were developed for discussion at stage III.

### Stage III: post-interview stakeholder focus group and goal-setting

Following presentation of maps and completion of worksheets, the highest-ranked problems were targeted for intervention and a projected timeline was assigned. The consensual goals were:

- to ensure efficient and comprehensive handover between liaison psychiatry and emergency department staff following psychiatry assessments (targets i, ii, xiii, xiv)
- to facilitate emergency department staff accessing the liaison psychiatry team for updates at morning shift-change (09.00 h) (targets i, ii, iii, xiii, xiv, xvii)
- to refine the referral pathway (emergency department to psychiatry) in order to clarify the role of each individual and the appropriate timing of referrals and to prevent staff making repeated and redundant contacts about the same patient (targets v, vii, viii, ix, x, xi)
- to establish a definition of ‘medical screening’, the process of medical assessment before psychiatry referral (targets vii, x, xi)
- to prevent or effectively manage acute agitation in psychiatric patients (targets v, vi)
- to formalise the role of security staff in the management of psychiatric patients, including education about statutory obligations and limitations (targets vi, xv).

### Stage IV: assessment of outcomes

#### Length of stay

A statistically significant Shapiro–Wilk test indicated a non-normal distribution of data (2017 *P* < 0.001; 2018 *P* < 0.001), indicating suitability for non-parametric analysis. There was a statistically significant improvement in the median length of stay between the pre-mapping group and the post-mapping group (median difference: 1 h; *P* = 0.015). The median length of stay pre-mapping was 8 h (interquartile range, IQR = 8) and post-mapping was 7 h (IQR = 7). There was a particular improvement in the number of psychiatric patients spending over 24 h in the department: length of stay exceeded 24 h for 5% of psychiatric patients in the pre-mapping group and 2% in the post-mapping group.

#### Attainment of goals

Five of the six targets were attained to the satisfaction of stakeholders, four on the projected timeline and one on a delayed timeline (Table 1).

One of the targets – ‘define medical screening’ – was revised and excluded at a subsequent clinical meeting, after concerns were raised that the implementation of such a definition could lead to a rigid clinical approach to screening psychiatric patients.

## Discussion

Process mapping, a component of lean management (‘lean’), is one of a number of management tools that aim to improve efficiency and eliminate ‘waste’.^13^ Lean was originally applied to the motor industry in Japan but the underlying philosophy lends itself to many types of organisation, including healthcare. Lean scrutinises and evaluates each component of a process so that ineffective, inefficient or potentially harmful elements (‘waste’) can be fixed or discarded.

Lean processes have been applied to other healthcare services, including ambulatory care settings^1^ and interventional radiology.^2^ Process mapping has been successful in these settings in identifying problems, reducing errors and generally improving efficiency; however, the outcomes in most of the previous studies have taken a qualitative approach rather than quantitative. Some studies have measured patient satisfaction before and after implementation of this method, but found no statistically significant difference.^14^ To our knowledge, the benefits of this method have not been studied in liaison psychiatry. The differences that exist between liaison psychiatry and other services, in particular the inherent unpredictability of an emergency department liaison psychiatry service, make it a unique setting that warrants particular attention. Lean methods have been applied to acute emergency settings^3^ and behavioural health crisis settings,^4^ both more similar to our service, but those studies did not assess the function of an emergency psychiatry service within a general emergency department, as is commonly the setting for the provision of emergency psychiatric care in Ireland and the UK. One such study of a stand-alone crisis centre found a significant improvement in door-to-door dwell time, but, as a disparate service to ours, without comparable interdisciplinary challenges, the findings are difficult to relate to a hospital setting.^4^

The use of process mapping in the present study afforded us the opportunity to visualise the journey of the psychiatric patient as they interacted with each individual stakeholder (Fig. 1) and as they were processed through the interdisciplinary department (Fig. 2). Doing so, we were able to set realistic, practical, timely and finite goals, thereby measurably improving efficiency. Further to this, we hoped that this process might help to improve the quality of working relationships between individuals and departments.

Lengthy waiting times of mentally ill patients in an emergency department exert stress on resources and increase the risk of adverse incidents.^9^ Before undertaking this quality improvement project, their median length of stay in this department (8 h) significantly exceeded the national target of 6 h. The National Emergency Medicine Programme in the jurisdiction of Ireland aims to ensure that 95% of patients are processed within 6 h.^15^ Process mapping and the series of interventions that followed led to a significant reduction in length of stay (median 7 h), with a particular reduction in the number of lengthy waiting times (5% exceeding 24 h pre-mapping, 2% exceeding 24 h post-mapping). This outcome, we postulate, was achieved by improving staff relations and agreeing on a series of low-burden and low-cost practical changes.

One such practical change was the management and prevention of acute agitation in mentally ill patients in the emergency department. The application of process maps enabled the stakeholder group to deconstruct the chain of events leading up to acute agitation:

- delayed handover following psychiatric assessment caused ambiguity about management (Table 1, proposal (a))
- psychiatric registrars reported being unable to identify the appropriate person in the emergency department to receive handovers (Table 1, proposals (a) and (b))
- the head of emergency department nursing observed that delays in administration of oral medication in the early stages of behavioural disturbance precipitated escalation of the behavioural disturbance, requiring emergency administration of intramuscular medication (Table 1; proposal (e))
- healthcare assistants observed that long periods spent in the contained environment of the emergency department led to patients becoming more irritable, but the assistants did not feel equipped to supervise time out of the department
- security staff were willing to supervise breaks with the healthcare assistant, but were unclear about their legal obligations and safeguards in relation to restraining and detaining patients (Table 1; proposal (f)).

Consensus on such solutions could not have been reached in the absence of this process, as non-clinical security staff and healthcare assistants – who provided important information and insights that were key to implementing solutions – are not routinely consulted by senior clinical and management staff. These types of solution, especially in cases such as this, reduce risks to patients and staff and reduce the burden on resources, thereby allowing the department to run more efficiently.

Further to these measurable benefits, process mapping facilitated progression from silo working to a cohesive team approach. The phenomenon of silo working leaves individual staff members feeling isolated and unsure about what to do or where to find help,^11^ especially in highly stressful situations such as a patient's acute agitation. Clarification of departmental policies and pathways, paired with improved individual flexibility and collegiality, empowered individuals to navigate difficult situations as a team so that interdisciplinary solutions were generated with minimal conflict. Process mapping provided a structure for this conversation to take place, thereby enhancing collegiality and collaborative care.

### Limitations

For process mapping to be successful in creating a consensus of opinion, the interviewer must remain objective. This process-mapping exercise was led by a senior registrar on the psychiatry team. Ideally, the lead role would be undertaken by an external participant, to avoid introducing interviewer bias (or the perception of interviewer bias by the stakeholders). This was not possible within the limits of our resources. This did not emerge as an obstacle in this study, probably because of the considerable trust between the specialties, but an independent interviewer would be essential if relationships were more fractious.

Compounding this, participants in this project were vulnerable to recall and reporting bias due to the retrospective nature of the interviews and the fact that they were asked to consider the pathway common to the majority of psychiatric cases, rather than specific cases.

Having evaluated the success of this process, it appears that neither of these limitations was prohibitive.

### Recommendations for utilising process maps in healthcare

#### Adhering to structure

We found that both the interviewer and stakeholders were tempted to offer personal conclusions and suggestions in stage I, which could have led to individual maps being contaminated by an individual's personal agenda. It was important throughout this process to remind the stakeholders that any useful solutions must be raised through the focus-group meeting in stages II and III.

#### Completing the full process

Change management requires energy and motivation and we found that sustaining momentum was a struggle at times. Process mapping does not serve its function if it ends after stage I, so persistence through stages II and III is needed to enable meaningful change to be made. It can be a challenge to convince busy professionals to sacrifice valuable time, but the value of participation en masse cannot be matched by only one or two people. Active involvement of consultants and heads of department from the outset is vital.

#### A word of caution

Not all situations are amenable to process mapping, so this method should be carefully considered before applying it to a problem situation.

Process mapping is a tool developed to examine a process, not a population. It is not designed to mediate interpersonal conflicts. Although we observed an improved sense of trust and collegiality, process mapping cannot enhance trust in a relationship where none exists.

The problems described herein are particular to our liaison psychiatry service, and may not mirror the challenges faced by other specialties or services; however, the general challenges encountered in the day-to-day provision of healthcare (e.g. inefficient use of resources and a tendency to resort to silo working) are ubiquitous across all settings. We have identified a method of overcoming these pitfalls. This experience can provide a blueprint for undertaking this kind of work in other fields of healthcare. We have found it a useful tool for enhancing working relationships and implementing immediate, lasting and meaningful change.

## About the authors

**Lauren Alexander**, MB BCh, BAO, MRCPsych, is a senior registrar in St Vincent's Psychiatric Hospital, Dublin; a postgraduate clinical research registrar in the Department of Psychiatry, St Vincent's University Hospital, Dublin; and an MD candidate at University College, Dublin. **Susan Moore**, MB BCh, BAO, MRCPsych, is a consultant in liaison psychiatry in the Department of Liaison Psychiatry, St Vincent's University Hospital, Dublin. **Nigel Salter**, MB BCh, BAO, FRCP, FRCEM, is a consultant in emergency medicine in the Emergency Department, St Vincent's University Hospital, Dublin. **Leonard Douglas**, MB BCh, BAO, MRCPsych, is a consultant in old age psychiatry in the Department of Old Age Psychiatry, St Vincent's University Hospital and is based at Carew House, Dublin, Ireland.
